# The Diagnostic and Prognostic Accuracy of Five Markers of Serious Bacterial Infection in Malawian Children with Signs of Severe Infection

**DOI:** 10.1371/journal.pone.0006621

**Published:** 2009-08-13

**Authors:** Enitan D. Carrol, Limangeni A. Mankhambo, Graham Jeffers, Deborah Parker, Malcolm Guiver, Paul Newland, Daniel L. Banda, The IPD Study Group, Elizabeth M. Molyneux, Robert S. Heyderman, Malcolm E. Molyneux, C. Anthony Hart

**Affiliations:** 1 Malawi-Liverpool-Wellcome Trust Clinical Research Programme, Blantyre, Malawi; 2 Department of Paediatrics, College of Medicine, University of Malawi, Zomba, Malawi; 3 Institute for Lung Health Clinical Sciences, Glenfield Hospital, Leicester, United Kingdom; 4 Health Protection Agency, Manchester Medical Microbiology Partnership, Manchester, United Kingdom; 5 Biochemistry Department, Alder Hey Children's NHS Foundation Trust, Liverpool, United Kingdom; 6 Division of Child Health, The University of Liverpool, Institute of Child Health, Alder Hey Children's NHS Foundation Trust, Liverpool, United Kingdom; 7 Division of Medical Microbiology, The University of Liverpool, Liverpool, United Kingdom; Columbia University, United States of America

## Abstract

**Background:**

Early recognition and prompt and appropriate antibiotic treatment can significantly reduce mortality from serious bacterial infections (SBI). The aim of this study was to evaluate the utility of five markers of infection: C-reactive protein (CRP), procalcitonin (PCT), soluble triggering receptor expressed on myeloid cells-1 (sTREM-1), CD163 and high mobility group box-1 (HMGB1), as markers of SBI in severely ill Malawian children.

**Methodology and Principal Findings:**

Children presenting with a signs of meningitis (n = 282) or pneumonia (n = 95), were prospectively recruited. Plasma samples were taken on admission for CRP, PCT, sTREM-1 CD163 and HMGB1 and the performance characteristics of each test to diagnose SBI and to predict mortality were determined. Of 377 children, 279 (74%) had SBI and 83 (22%) died. Plasma CRP, PCT, CD163 and HMGB1 and were higher in HIV-infected children than in HIV-uninfected children (p<0.01). In HIV-infected children, CRP and PCT were higher in children with SBI compared to those with no detectable bacterial infection (p<0.0005), and PCT and CD163 were higher in non-survivors (p = 0.001, p = 0.05 respectively). In HIV-uninfected children, CRP and PCT were also higher in children with SBI compared to those with no detectable bacterial infection (p<0.0005), and CD163 was higher in non-survivors (p = 0.05). The best predictors of SBI were CRP and PCT, and areas under the curve (AUCs) were 0.81 (95% CI 0.73–0.89) and 0.86 (95% CI 0.79–0.92) respectively. The best marker for predicting death was PCT, AUC 0.61 (95% CI 0.50–0.71).

**Conclusions:**

Admission PCT and CRP are useful markers of invasive bacterial infection in severely ill African children. The study of these markers using rapid tests in a less selected cohort would be important in this setting.

## Introduction

Serious bacterial infection (SBI) is a major cause of morbidity and mortality in children in the developing world and is responsible for about 60% of childhood mortality. Community-acquired bacteraemia has been demonstrated to be a major cause of death in children admitted to a rural hospital in Kenya, accounting for a quarter of all in-hospital deaths [1]. Early recognition and appropriate antibiotic treatment can significantly reduce morbidity and mortality [2], [3].

Various diagnostic markers of sepsis have been suggested to facilitate early diagnosis of serious bacterial infection (SBI) and to inform prognosis, in a variety of clinical settings in different patient groups. These markers include C-reactive protein (CRP), procalcitonin (PCT), soluble triggering receptor expressed on myeloid cells-1 (sTREM-1), haemoglobin scavenger receptor (CD163) and High Mobility Group Box 1 (HMGB1), but results have been inconsistent and variable depending on the selection criteria of patients [4], [5], [6], [7]. Procalcitonin (PCT) levels begin to increase 3–4 hours after injection of an endotoxin stimulus in human subjects, peak at about 6 hours and then plateau for up to 24 hours [8]. In contrast, C-reactive protein (CRP) levels rise between 12 and 18 hours after bacterial challenge [8].

TREM-1 is a cell-surface receptor and its expression is up-regulated on phagocytic cells in the presence of bacteria and fungi. TREM-1 is shed from the membrane of activated phagocytes and is present in a soluble form, sTREM-1, in body fluids. TREM-1 expression on monocytes is significantly higher in septic shock patients than non-septic patients [9].

Haemoglobin scavenger receptor (CD163) is exclusively expressed in the monocyte-macrophage cell lineage. It is involved in endocytosis of haptoglobin-haemoglobin complexes. Soluble haemoglobin scavenger receptor (sCD163) is a product shed from the monocyte-macrophage membrane. Increased plasma levels of sCD163 have been observed in patients with severe sepsis [4], bacterial meningitis [10], and bacteraemia [11]. sCD163 was found to be a reliable predictor of a fatal outcome in adult patients with pneumococcal bacteraemia [12].

HMGB1, a nuclear protein, is a delayed mediator of inflammation, with release occurring about 12–18 hours after classical early pro-inflammatory mediators such as TNF and IL-1. High levels of HMGB1 are seen in severe sepsis and septic shock, with levels remaining high in non-survivors [13], [7].

The mortality from community acquired bacteraemia among children in sub-Saharan Africa is significant, with a third of deaths occurring on the day of admission and two thirds within two days [1]. What is needed is a rapid and reliable bedside test that accurately predicts SBI, and opens up an opportunity to either initiate antibiotics early or to identify children at highest risk of death in whom second line antibiotics or aggressive fluid therapy might be targeted. PCT and CRP have been evaluated in the developing world setting [14], [15], [16] but the assessment of sTREM-1, CD163 and HMGB1 as markers of SBI has never previously been performed in African children. The aim of this study was to evaluate the diagnostic and prognostic utility of these markers in diagnosing SBI in Malawian children presenting to the accident and emergency department with signs and symptoms suggestive of meningitis or pneumonia.

## Methods

We prospectively enrolled children between April 2004 and October 2006 who presented to the Accident and Emergency Department and the Admissions Unit of Queen Elizabeth Central Hospital, Blantyre, Southern Malawi, which serves a population of approximately one million. This is a government-funded teaching and referral hospital with 150 paediatric beds, although paediatric inpatients are often in excess of 300. Children aged 2 months to 16 years identified as possible cases of pneumonia (respiratory rate≥50/min for children <12 months and ≥40/min for children ≥12 months) or meningitis (stiff neck, bulging fontanelle, fever and convulsions) were consecutively recruited into the study. Children with significant co-existing co-morbidities (eg congenital heart disease, chronic lung disease, end-stage AIDS) were excluded. *Haemophilus influenzae* b vaccination is administered routinely in the childhood immunisation schedule in Malawi.

The primary outcome measure was bacteriological confirmation of infection (SBI) and the secondary outcome measure was death/survival in hospital, Afebrile children, without malaria parasitaemia, from the same villages as the cases, were used as controls. All controls were HIV-uninfected.

Ethical approval for this study was granted from The College of Medicine Research Committee (COMREC), Malawi and The Liverpool School of Tropical Medicine Research Ethics Committee. Parents or guardians gave written informed consent for children to enter the study.

### Definitions

#### Cases (n = 377)

Children who presented with signs and symptoms of meningitis or pneumonia.

#### Bacterial pneumonia (n = 95)

Clinical signs of pneumonia (cough, fever, crackles, bronchial breath sounds) and radiological evidence of pneumonia (focal or lobar consolidation).

#### Bacterial meningitis (n =  282)

Clinical signs of meningitis, and abnormal CSF cell count, >10 cells/µl.

#### Serious bacterial infection (SBI+ve) (n = 280)

Children who presented with either bacterial meningitis or bacterial pneumonia, in whom a bacterial pathogen was identified by culture, polysaccharide antigen test or PCR (*Streptococcus pneumoniae, Neisseria meningitidis, and Haemophilus influenzae b*).

#### No detectable bacterial infection (SBI-ve) (n = 97)

Children who presented with bacterial meningitis or bacterial pneumonia, but who were negative for any bacteria on culture, polysaccharide antigen test or PCR (*S.pneumoniae, N.meningitidis, and H. Influenzae b*).

#### Invasive pneumococcal disease (IPD) (n = 230)

Children with SBI in whom *S. pneumoniae* was identified (by culture, microscopy, antigen testing or PCR) from one or more of the following normally sterile body sites: blood, cerebrospinal fluid, lung aspirate.

### Microbiological methods

For blood culture, 1–2 mls of venous blood was drawn after swabbing the venepuncture site with alcohol. When this sample was taken at the same time as the other study blood samples, the blood culture bottle was inoculated first. The BacT/lert 3D automated system was used (BioMerieux), and isolates were identified using standard techniques[17]. Diphtheroids, micrococci, *Bacillus species*, Coagulase-negative staphylococci and mixed growth of skin flora were considered to be contaminants.

CSF samples were examined microscopically for total white cell count and differential. Gram stain was performed on all samples that were turbid or had >8 white cells/µL. After centrifugation, deposits were cultured on sheep blood agar and incubated in a candle jar at 37 °C for 48 h. 5 ml brain-heart infusion broth with 1% Vitox (Oxoid, Basingstoke, UK) was added to the remaining deposit for enrichment culture. This broth was incubated for 48 h and the centrifuged deposit was cultured onto sheep blood agar, which was also incubated for 48 h. Latex agglutination tests using the Wellcogen bacterial antigen kit (Abbott Murex Biotech, Dartford, UK) were performed on CSF samples suggestive of meningitis, but with negative Gram staining.

All blood and CSF samples were assayed using a multiplex PCR assay for the detection of *N. meningitidis*, *H. influenzae* and *S. pneumoniae* as previously described [18]. The PCR assay for the quantification of pneumococcal DNA has been described previously [19].

### Malaria diagnosis

Microscopic diagnosis was based on best standard practice of district-hospital and health-centre general laboratories in sub-Saharan Africa. In brief, blood films were stained with Fields stain and parasite densities estimated from thick films by counting the number of parasites per 200 white blood cells (WBC) assuming a total count of 8000/ml.

### CRP and PCT assay

The assays for CRP and PCT measurement have previously been described [20].

### TREM-1, CD163 and HMGB1 ELISAs

TREM-1, CD163 and HMGB1 were measured using a commercially available sandwich enzyme immunoassays according to the manufacturer's instructions (TREM-1: Quantikine, R&D Systems, Minneapolis, USA, CD163: Bachem (UK), St Helens, Merseyside, UK, and HMGB1: Shino-test, Kanagawa, Japan).

### Cytokine determination

Cytokine determination was performed in plasma using a commercial 27-plex Bioplex Cytokine kit (Bio-Rad Laboratories Inc, California, USA) that utilises Luminex 100 technology in the Bio-plex Protein Array System according to the manufacturer's instructions (Bio-Rad Laboratories Inc, California, USA).

### Statistical analysis

**Figure 1:**
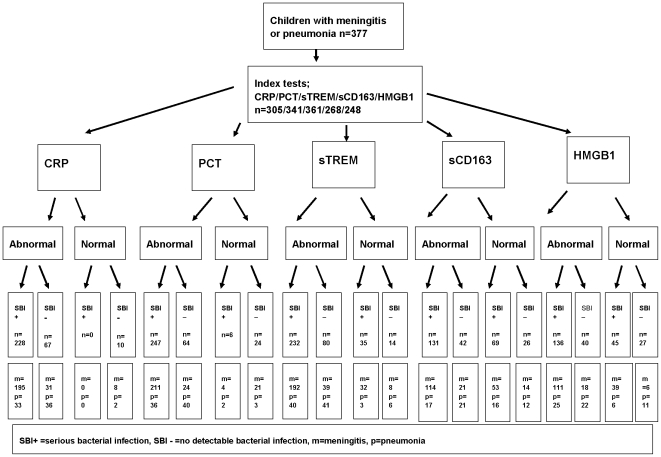
Flow diagram showing the number of patients undergoing index tests and the number of patients with SBI and meningitis and pneumonia, according to STARD guidelines.

Statistical analysis was performed using SPSS for Windows, version 15.0, (Illinois, USA). The data when plotted did not follow a normal distribution, and therefore the Mann Whitney Test was used to compare distributions, and Spearman's correlation coefficient for correlations of clinical and laboratory variables. The General Linear Model was used to explore the effects of confounding factors (HIV status, neutrophil count, age, sex, duration of symptoms and previous antibiotic administration) on comparison groups. Cut-off values to determine “normal” and “abnormal” values (Figure 1) were derived from previous published studies and the following were used; CRP≤10 mg/l, PCT≤0.5 ng/ml, TREM-1≤25 ng/ml, CD163≤5000 ng/ml and HMGB1≤5 ng/ml [7], [10], [21]. Receiver operator characteristic (ROC) curves were used to determine the areas under the curve (AUCs) with 95% confidence intervals for the five markers to predict SBI and death. The laboratory assays for bacteriological confirmation and the markers of infection were performed by investigators blinded to the clinical data. The clinicians involved in managing the cases were not involved in performing any of the laboratory assays.

The study was reported according to STARD guidelines which includes method of recruitment of patients, orders of test execution, and numbers of patients undergoing the tests under evaluation and the numbers of patients with the reference standard [22], [23] (Figure 1).

## Results

### Patient characteristics

There were 377 children who presented with signs of pneumonia (n = 95) or meningitis (n = 282). In the cases, there were 215 males (57%) and median age was 2.3 years, interquartile range (IQR) (0.8 to 6.1 years). In the controls there were 13 males (87%) and median age was 10.0 years (IQR 6 to 13 years). Of the cases, 190 (50.4%) were HIV-infected, and none of the controls were HIV-infected. Overall, 83 children (22%) died. In 13/360 children, the blood film was positive for malaria parasites. In these 13 children, CRP, s-TREM-1, CD163 and HMGB1 were higher than in those negative for malaria parasites, but these differences were not significant. Of the children with malaria parasitaemia, 2 children had 4+ parasitaemia, 1 had 3+, 2 had 2+, and 8 had 1+. In 5 of the children with parasitaemia, blood cultures were also positive; *S. pneumoniae*: 3 children had 1+ and 1child had 2+ parasitaemia, and *Salmonella* Enteritidis: 1 child had 1+ parasitaemia.

**Figure 2:**
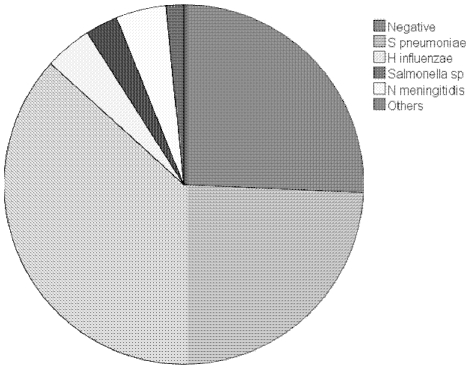
Pie chart showing aetiology of serious bacterial infection.

A total of 280 children (74%) had serious bacterial infection (SBI+ve), of which 131 (47%) were blood culture positive, 144 (52%) were HIV-infected, and 67 (24%) died. Table 1 compares the baseline patient characteristics in the SBI+ve and SBI-ve groups. The predominant pathogen causing disease was *S. pneumoniae* (n = 230, 61%), followed by *N. meningitidis* (n = 17, 4.5%), *H. influenzae* (Hib n = 16, non- Hib n = 4, total 4.2%) and *Salmonella enterica* (*S. enterica* serovar Typhimurium n = 9, *S. enterica* serovar Enteritidis n = 2, total: 2.9%). In 97 children (26%) no pathogen was identified (Figure 2). In the SBI-ve group, 46 (47%) were HIV-infected and 16 (16%) died. Blood culture positivity was reduced in those who reported previous antibiotic use (33% versus 67%, p = 0.004).

**Table 1:** Characteristics of children with serious bacterial infection (SBI) and those with no bacterial infection (NBI) detected> Numeric value are median and interquartile range (IQR).

	SBI (n = 280)	NBI (n = 97)	p value
Age (years)	2.0	2.5	0.77
*median(IQR)*	(0.6–6.9)	(1.0–5.7)	
Males	154 (55%)	61 (63%)	0.18
Duration of symptoms (days) *median(IQR)*	3 (2–5)	3 (2 – 5)	0.76
Previous antibiotics (%)	123 (44%)	37 (38%)	0.32
Meningitis	235 (84%)	47 (49%)	<0.0005
Mortality (%)	68 (24%)	15 (15%)	0.07
HIV-infected (%)	145 (52%)	45 (47%)	0.41
Height for age Z score <−3 (severe stunting) (%)	42/272 (15%)	16/91 (18%)	0.63
Weight for height Z score <−3 (severe wasting) (%)	37/218 (17%)	11/82 (13%)	0.45
White cell count (WCC)×10^9^/l	12.6	13.8	0.09
*median(IQR)*	(7.8–20.0)	(10.0–20.4)	
Neutrophil count×10^9^/l	10.6	8.9	0.78
*median(IQR)*	(4.6–16.3)	(5.5–15.5)	

### CRP, PCT, s-TREM-1, CD163 and HMGB1 in HIV-infected and HIV-uninfected children

Median CRP, PCT, s-TREM-1, CD163 and HMGB1 values were higher in HIV-infected compared to HIV-uninfected cases (277.3 versus 239.0 mg/l, 34.4 versus 16.5 ng/ml, 52.8 versus 61.6 ng/ml, 6880.7 versus 5329.8 ng/ml and 7.0 versus 5.8 ng/ml, p = 0.007, p<0.0005, p = 0.08, p = 0.001, p = 0.004, respectively).

### Subgroup comparisons of CRP, PCT, s-TREM-1, CD163 and HMGB1 in HIV-infected children

Median PCT values were significantly higher in bacterial meningitis compared to bacterial pneumonia, and median s-TREM-1 was significantly lower (Table 2). Median CRP, PCT, and HMGB1 values were significantly higher in SBI+ve compared to SBI-ve cases. Within SBI+ve cases, s-TREM-1 was significantly higher in infections other than IPD compared to IPD. Median PCT and CD163 values were significantly higher in non-survivors compared to survivors (Table 2).

**Table 2:** Comparison of median CRP, PCT, s-TREM-1, CD163 and HMGB1 values in different groups stratified by HIV status.

	HIV-infected					HIV-uninfected				
	CRP (mg/l)	PCT (ng/ml)	s-TREM-1 (ng/ml)	CD163 (ng/ml)	HMGB1 (ng/ml)	CRP (mg/l)	PCT (ng/ml)	s-TREM-1 (ng/ml)	CD163 (ng/ml)	HMGB1 (ng/ml)
Meningitis	279	44	50	7144	6.8	232	18	61	5430	5.8
Pneumonia	276	13	64	5449	7.6	275	8	64	5219	5.5
	*p = NS*	*p = 0.003*	*p = 0.01*	*p = 0.06*	*p = NS*	*p = NS*	*p = NS*	*p = NS*	*p = NS*	*p = NS*
SBI+ve	291	46	53	6900	7.6	253	22	65	5324	5.9
SBI–ve	135	3	54	5544	6.0	127	4	57	5601	4.9
	*p<0.0005*	*p<0.0005*	*p = NS*	*p = NS*	*p = 0.05*	*p<0.0005*	*p<0.0005*	*p = NS*	*p = NS*	*p = NS*
IPD	294	48	52	6948	7.6	277	25	57	5324	5.8
SBI(other than IPD)	279	41	70	6160	6.9	247	55	114	5549	6.5
	*p = NS*	*p = NS*	*p = 0.02*	*p = NS*	*p = NS*	*p<0.0005*	*p = NS*	*p = 0.01*	*p = NS*	*p = NS*
Survivor	280	24	54	5783	6.9	247	14	61	5243	5.6
Non-survivor	256	90	52	7972	7.7	201	19	68	6308	6.5
	*p = NS*	*p = 0.001*	*p = NS*	*p = 0.05*	*p = NS*	*p = NS*	*p = NS*	*p = NS*	*p = 0.05*	*P = NS*

(NS = non-significant).

### Sub group comparisons of CRP, PCT, s-TREM-1, CD163 and HMGB1 in HIV-uninfected children

There was no significant difference in median CRP, PCT, s-TREM-1, CD163 and HMGB1 values between bacterial meningitis compared to bacterial pneumonia. Median CRP and PCT values were significantly higher in SBI+ve compared to SBI-ve cases. Within SBI+ve cases, s-TREM-1 was significantly higher in infections other than IPD compared to IPD, and CRP was significantly higher in IPD compare to infections other than IPD. Median CD163 values were significantly higher in non-survivors compared to survivors (Table 2).

### Correlations between CRP, PCT, s-TREM-1, CD163 and HMGB1 and pro- and anti- inflammatory cytokines

There were weak but significant correlations between the five markers of infection and the pro- and anti-inflammatory cytokines, interleukin-6 (IL-6), interleukin-8 (IL-8), interleukin-1Ra (IL-1Ra) and interleukin-10 (IL-10). These are shown in Table 3.

**Table 3:** Correlation between markers of infection and pro-and anti- inflammatory cytokines (Spearman's correlation coefficient. NS = non-significant).

	Log IL-6	Log IL-8	Log IL-1Ra	Log IL-10
CRP	0.38	0.14	0.33	NS
	p<0.0005	p = 0.04	p<0.0005	
PCT	0.62	0.51	0.63	0.32
	p<0.0005	p<0.0005	p<0.0005	p<0.0005
sTREM-1	NS	NS	NS	NS
CD163	0.23	0.15	0.23	NS
	p<0.0005	p = 0.02	p<0.0005	
HMGB1	0.25	0.26	0.21	0.23
	p<0.0005	p<0.0005	p = 0.001	p<0.0005

### Discriminatory power of CRP, PCT, s-TREM-1, CD163 and HMGB1 in predicting SBI or death using Receiver Operator Characteristic (ROC) curves

**Figure 3:**
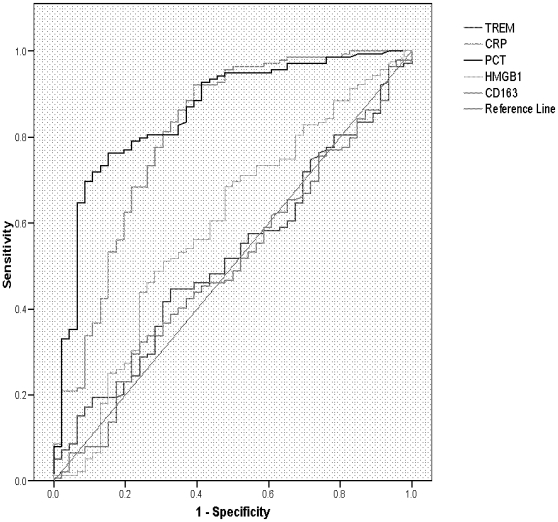
ROC curve of CRP, PCT, s-TREM-1, CD163 and HMGB1 as markers of SBI.

**Figure 4:**
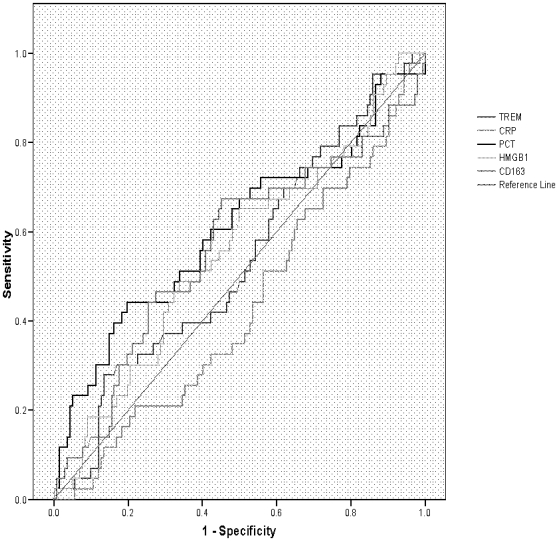
ROC plot of CRP, PCT, s-TREM-1, CD163 and HMGB1 as predictors of mortality.

The areas under the ROC curve (AUCs) for CRP, PCT, sTREM-1, CD163 and HMGB1 in predicting SBI were 0.81 (95% CI 0.73–0.89), 0.86 (95% CI 0.79–0.92) and 0.52 (95% CI 0.43–0.61), 0.50 (95% CI 0.41–0.60) and 0.59 (95% CI 0.50–0.69) respectively. The AUCs for CRP, PCT, sTREM-1, CD163 and HMGB1 in predicting death were 0.43 (95% CI 0.33–0.53), 0.61 (95% CI 0.50–0.71) and 0.54 (95% CI 0.44–0.64), 0.56 (95% CI 0.46–0.67) and 0.56 (95% CI 0.46–0.65) respectively (Figures 3 and 4).

## Discussion

We have shown that PCT is the best diagnostic marker of SBI in Malawian children presenting with signs of severe sepsis. Although none of the markers demonstrated any value in predicting death, PCT was also the best prognostic marker in children with severe sepsis. PCT and CRP were significantly increased in SBI, and these differences remain significant when stratified by HIV status. CD163 is significantly increased in non-survivors, and PCT is significantly increased in HIV-infected survivors. Although the performance of CRP was similar to that of PCT in predicting SBI, the rapid kinetics of PCT compared to CRP make it more likely to detect sepsis early, and could be used to initiate antibiotics early in children at risk of sepsis. Our data are consistent with those of Palmer in which CRP did not show enough sensitivity and specificity to be used alone as a predictor of SBI in infants [14], and the study by Madhi where CRP and PCT improved the diagnosis of radiological pneumonia [16]. In contrast, a study by Cheung found that CRP was a better discriminator of pneumonia than PCT [15]. Our findings also support those of Moller [12], which showed that CD163 was superior to other markers in predicting fatal outcome in adults with pneumococcal bacteraemia.

It is inconceivable that clinicians would ever withhold parenteral antibiotics from children with signs of pneumonia or meningitis in a setting where a quarter of children die, and where diagnostic facilities are not optimal. However, our study suggests that in children who would not necessarily receive parenteral antibiotics (i.e. sick enough to be admitted to hospital but not meeting WHO syndromic criteria for parenteral antibiotics), the availability of a rapid PCT test could be clinically very useful.

Strategies to reduce the global burden of sepsis include prevention through immunization, early recognition and treatment and development of new diagnostics and therapeutics[3]. Our study suggests that a rapid test for PCT could help to identify children with SBI but without signs of severe sepsis, and children in whom aggressive intravenous fluid resuscitation and adjunctive therapies such as low-dose hydrocortisone, might be targeted. Our study also suggests that PCT could potentially be used to define disease severity objectively for the purpose of comparing groups in pathogenesis studies, and to define patients who might be eligible for randomised controlled trials of new therapies in bacterial sepsis.

A semi-quantitative immunochromatographic bedside test for PCT is currently available (Brahms Diagnostica GMBH, Berlin) [24], however, this test is limited by lack of sensitivity. A newer, more sensitive bedside assay which uses whole blood, is in development (PCT Direct, Brahms Diagnostica GMBH, Berlin), with a sensitivity of 0.2 ng/ml and a range up to 7 ng/ml. It will use a dedicated PCT reader and is expected to be launched in 2010. This rapid test, if available, could allow PCT to be used in resource poor settings, as a point-of-care test, to direct clinical decision making. The success of rapid diagnostic tests in resource-poor settings has been illustrated by their use the diagnosis of HIV and malaria. In the study from rural Kenya, the largest such study ever to be conducted in sub-Saharan Africa, deaths in children with bacteraemia were rapid, with 33% occurring on the day of admission, and 70% within two days [1]. This considerable mortality, within a short interval despite a high standard of care, highlights the need for rapid and reliable diagnostic tests such as PCT in this setting.

The strengths of this study are that we studied a large number of children in whom the clinical phenotype was well described, and in whom we investigated for the presence of bacterial infection with culture, antigen testing and PCR. Our study has some limitations; we studied a selected group of patients (with symptoms and signs of meningitis or pneumonia), we measured the markers at one time point only, and we did not investigate the membrane-bound components of CD163 or TREM-1. Data from other African studies suggest that both malaria and tuberculosis may increase levels of CD163 [25], [26], but our study did not demonstrate that malaria co-infection may significantly influence levels of these markers in acute bacterial infections. A case-control study design may potentially over-estimate the predictive value of a test, but we have attempted to eliminate further bias by comparing the tests using an independent reference standard (microbiologically confirmed disease) according to STARD guidelines.

Before we can recommend PCT for more widespread use, we recommend that the study is repeated in a less selected cohort of all children presenting with signs suggestive of bacterial infection, and that a randomised controlled trial (RCT) of PCT be conducted to assess the potential for PCT-guided management to reduce mortality. Recent studies of RCTs of PCT-guided therapy in ICU patients have suggested that PCT-guided decision making may reduce antibiotic use and shorten PICU stay [27], [28].

In conclusion, we have shown that among the five markers tested, PCT is the best diagnostic and prognostic marker of SBI in Malawian children with severe sepsis, including those with HIV infection. This study provides valuable new information about the performance of these markers in a developing world setting, and suggests that the study needs to be repeated in a less selected cohort.
